# The Effects of Different Ionic Liquid Coatings and the Length of Alkyl Chain on Antimicrobial and Cytotoxic Properties of Silver Nanoparticles 

**DOI:** 10.22037/iej.v12i4.17905

**Published:** 2017

**Authors:** Abbas Abbaszadegan, Ahmad Gholami, Sara Abbaszadegan, Zeynab Sadat Aleyasin, Yasamin Ghahramani, Samira Dorostkar, Bahram Hemmateenejad, Younes Ghasemi, Hashem Sharghi

**Affiliations:** a *Department of Endodontics, Dental School, Shiraz University of Medical Sciences, Shiraz, Iran; *; b *Pharmaceutical Sciences Research Center and Department of Pharmaceutical Biotechnology, School of Pharmacy, Shiraz University of Medical Sciences, Shiraz, Iran; *; c *Dental School, Shiraz University of Medical Sciences, Shiraz, Iran ; *; d *Department of Oral Medicine, Dental School, Shiraz University of Medical Sciences, Shiraz, Iran ; *; e *Department of Chemistry, Shiraz University, Shiraz, Iran*

**Keywords:** Antibacterial Agents, Chlorhexidine, Cytotoxicity, Metal Nanoparticles, Sodium Hypochlorite

## Abstract

**Introduction:**

The antibacterial efficacy and toxicity of silver nanoparticles (AgNPs) depends on their physicochemical properties including size, shape, surface charge and surface coatings. The Objectives of this study were: *i)* To synthesize and characterize positively charged AgNPs coated by different ionic-liquids with different alkyl chain lengths, *ii)* To evaluate the antimicrobial activity of these nanoparticles against *Enterococcus faecalis *compared to sodium hypochlorite (NaOCl) and chlorhexidine (CHX), *iii)* To compare the cytocompatibility of these solutions against L929 mouse fibroblasts.

**Methods and Materials:**

AgNPs with positive surface charges capped by two different ionic liquids [imidazolium (Im) and pyridinium (Py)] with two alkyl chain lengths (C_12_ and C_18_) were synthesized. Im and Py were also tested as control groups. The characterization revealed synthesis of spherical NPs in the size range of 6.7-18.5 nm with a surface charge ranging from +25 to +58 mV. To standardize the comparisons, the surface charge to radius ratio of each nanoparticle was calculated. The minimum inhibitory concentrations (MIC) of the AgNP solutions, NaOCl and CHX were determined against *E. faecalis *by a microdilution test. An MTT-based cytotoxicity assay evaluated the cytotoxicity of the solutions in different concentrations on L929 fibroblasts. One-way and two-way ANOVA were used for statistical analysis.

**Results:**

All tested AgNPs reached MIC_90_ in significantly lower concentrations compared to CHX and NaOCl. C_12 _Py-coated AgNPs had the lowest MIC_90_ value. CHX and NaOCl were more toxic on fibroblasts than all tested AgNPs. Im-coated AgNPs had better compatibility with fibroblasts than Py-coated particles; and C_12 _Im AgNPs had the best biocompatibility. Variations in alkyl chain length had no effects on the biocompatibility of AgNPs.

**Conclusion:**

Py improved the antibacterial efficacy of AgNPs compared to Im; however, it had a negative effect on cytocompatibility. Alkyl chain length had no effects on AgNPs’ bioactivity.

## Introduction

Silver is well known for its antibacterial properties in the field of medicine [1, 2]. As a result, over the past decade, many researchers have focused on the synthesis and application of nano scale of silver particles to obtain greater antibacterial activity [3, 4]. Therefore, these nanoparticles have drawn the attention in many fields including dentistry in to manufacturing many dental products such as antibacterial solutions, endodontic sealers, restorative materials and implants [5-7].

The antibacterial efficacy of silver nanoparticles (AgNPs) depends on their physicochemical properties including size, shape, surface charge and surface coatings [8-11]. Several reports have addressed the aggregation of AgNPs in suspension form as a problem that can lead to a reduced surface area and change the antimicrobial potency [12, 13]. To overcome this problem, coatings such as citrate, starch and different forms of ionic liquids are frequently used as capping agents. These capping agents are reported to change almost all properties of nanoparticles [14]. It is shown that AgNPs without surface modifiers or stabilizers had significant cytotoxic properties [15] while coated AgNPs are reported to have little to no cytotoxicity [16].

Ionic liquids are novel antibacterial salts [17] consisting of large cations such as imidazolium (Im), pyridinium (Py) and anions including chloride, bromide and nitrate. The large organic cations are bonded to an alkyl chain which alters the hydrophobicity of the ionic liquids. Changes in the cationic or anionic compartments and variations in the number or length of the alkyl chains, may alter the characteristics of the ionic liquids [18]. Earlier investigations revealed that increase in the alkyl chain length will influence the lipophilicity. Given that higher lipophilicity may result in easier penetration of AgNPs into the cell layer of prokaryotic or eukaryotic cells, it may influence the antibacterial activity and toxicity of the polymers [18-20]. Furthermore, the greater repulsion between AgNPs with longer alkyl chains may lead to an increase in inter-particle distances, decreasing aggregation and eventually promoting the efficacy of NPs [21]. Also, former investigations revealed that the microbial biofilms can be broken down by longer alkyl chains containing Im and Py compounds [22, 23].

Previously, we reported the synthesis of differently charged AgNPs and the effect of surface charge on their antibacterial and cytotoxic properties [14, 24]. We found that positively charged Im-based ionic liquid protected AgNPs had promising antibacterial results against *E. faecalis* and exhibited a high level of cytocompatibility with L929 fibroblasts.

The current investigation was designed to synthesize positively charged AgNPs capped by two different families of ionic liquids (Im and Py) with different alkyl chain lengths (C_12_ and C_18_) and to evaluate the antibacterial potency of these variations of nanosilver solutions against *E. faecalis *and their cytocompatibility on L929 mouse fibroblast cells compared to NaOCl and chlorhexidine (CHX) as two conventional endodontic disinfectant solutions.

## Materials and Methods

***Synthesis of the ionic liquids***

1-methylimidazole, pyridine, 1-chlorododecyl, 1-chloctadecane and diethyl ether were supplied from Merck (Darmstadt, Germany) or Fluka (Buchs, Switzerland) and used without any further purification. According to the previously reported procedure [25] ionic liquids were synthesized by reacting 1-methylimidazolium or pyridine with excess amount of 1-chlorododecane or 1-chlorooctane without any additional solvent in an around-bottomed flask fitted with a reflux condenser (heating and stirring at 70^o^C for 48-72 h). Then the viscous liquid was cooled to room temperature and was washed by diethyl ether. The resulting ionic liquids were 1-dodecyl-3-methylimidazolium chloride ([C_12_mim][22]), 1-octadecyl-3-methylimidazolium chloride ([C_18_mim][22]), 1-dodecyl pyridinium chloride ([C_12_Py][22]) and 1-octadecyl pyridinium chloride ([C_18_Py][22]).

***Synthesis of AgNPs***

The positively charged ionic liquid-protected AgNPs, were prepared according to the procedure described by Hemmateenejad *et al.* [26]. In brief; all glassware were placed in 1:3 HCl/HNO_3_ solution, rinsed with triply distilled water for three times. Then, 1.0 mL of 0.01M AgNO_3_ aqueous solution was added to 20 mL of 6.2 mM aqueous solution of each ionic liquid and the solution was stirred vigorously. Freshly-prepared 0.4 M NaBH_4_ aqueous solution was then added to the stirred solutions dropwise until the color of the solution became golden. Subsequently, the colloidal solutions were centrifuged for 20 min to remove excess ionic liquids. The formed golden-colored solution was stored at room temperature.

***Characterization of the AgNPs***

AgNP solutions were characterized with the help of ultrospec 3000 UV-Vis spectrophotometer (Biochrom Ltd, Cambridge, England) at a resolution of 1 nm. Moreover, for transmission electron microscopy (TEM) analysis, measurements were performed on the AgNP solutions operating at 200KV and the average sizes of 250 particles were recorded. Further, the surface charges of the synthesized nanoparticles were measured with the help of zeta potential analyzer (Zeta Plus, Brookhaven instruments, NY, USA).

***Preparation of the experimental solutions***

In this study, 6 experimental solutions were prepared as follows: Im- and Py-protected AgNP solutions with two different alkyl chains (C_12_, C_18_), 0.2% CHX (Sigma-Aldrich Co., St. Louis, MO, USA) with equivalent concentration of 4×10^-3 ^M/L (molecular weight=505.446 g/M) and 5% NaOCl (Sigma-Aldrich Co., St. Louis, MO, USA) with equivalent concentration of 0.67 M/L (molecular weight=74.44 g/M).

***Determination of minimum inhibitory concentration (MIC)***

Antibacterial activity of the experimental solutions was measured against planktonic *E. faecalis* strain AGH011 using MIC test. All experiments were performed in triplicate and according to the guidelines of the Clinical and Laboratory Standards Institute. In this regard, two-fold serial dilutions up to seven times were prepared for all solutions. Serial dilutions of each solution were poured into a 96-well microplate with Muller-Hinton Broth (MHB) medium supplemented with calcium (25 mg/L) and magnesium (12.5 mg/L) to obtain a final volume of 90 µL. Then, 10 µL of *E. faecalis* suspension (matching the turbidity of 2 McFarland standards) was added to each microplate and incubated for 24 h at 37^°^C. The optical density of the microplates was assessed using an ELISA reader (Biotek, Winooski, VT, USA) at the wavelength of 600 nm. The MIC_90 _was defined as the value which inhibited 90% of the bacterial growth when compared with control group growth. The negative and positive control groups were culture media and *E. faecalis* inoculated culture media respectively. Also, Ampicillin was used as a standard group. Also 50 L of Im and Py were tested as control groups.

**Figure 1:**
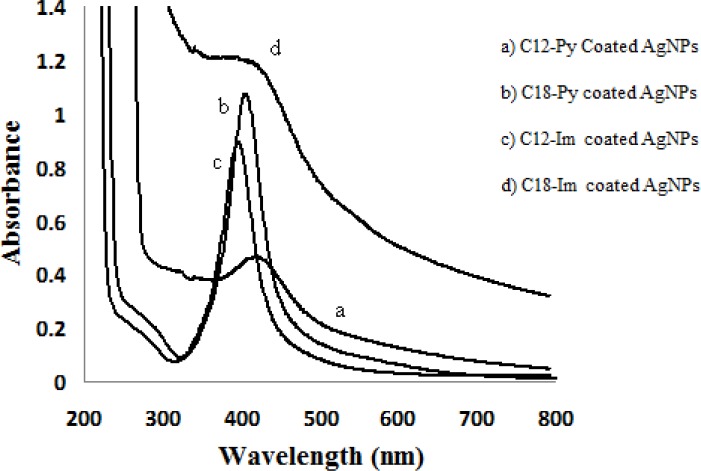
The UV-Visible spectra of synthesis of Ag NPs (Im: imidazolium, Py: pyridinium, C_12_: alkyl chain with 12 Carbons, C_18_: alkyl chain with 18 Carbons

***Cytocompatibility assessment***

Cytotoxicity of the experimental solutions was evaluated on L929 mouse fibroblast cells using MTT assay. Briefly, L929 mouse fibroblasts in PRMI1640 media were seeded into a 96-well cell culture plate and incubated in an atmosphere of 5% Co_2_ and 95% air at 37^°^C for 24 h. Then, media in each well was replaced by 100 µL of each experimental solutions dissolved in RPMI1640 and was incubated again at 37^°^C. After 24 h, 25 µL of MTT solution [3-(4, 5- dimethylthiazol-2-yl)-2,5-diphenyl tetrazolium bromide] (Sigma-Aldrich Co., St. Louis, MO, USA) was added to each well and incubated at the same atmosphere for 4 h. Afterwards, 100 µL of dimethyl sulfoxide (DMSO) was added to each well and incubated for 10 min. The absorption of solutions was read by an ELISA plate reader (Biotek, Winooski, VT, USA) at the wavelength of 540 nm. The culture media and 35% hydrogen peroxide were regarded as routine negative and positive control groups. Moreover, 50 L of Im and Py were also tested to remove the possible effect of these substances on cytotoxicity of AgNPs. 

All procedures were done in triplicate for each group. The mean cell viability values were expressed as percentage of negative control.

## Results

***Characterization of the AgNPs***

**Figure 2:**
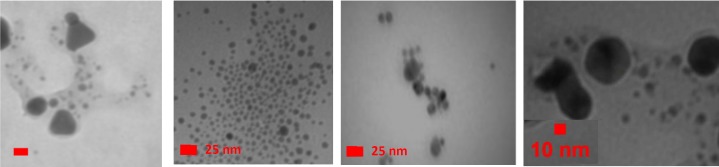
*TEM images of the synthesized AgNPs. *A)* C*_12 _*Pyridinium-coated;* B)* C*_18 _*Pyridinium-coated;* C)* C*_12 _*Imidazolium-coated;* D)* C*_18 _*Imidazolium-coated*

UV visible spectrophotometer characterization and transmission electron microscopy were carried out to characterize the properties of the synthesized AgNPs (Figure 1 and Table 1). Figure 2 demonstrates the transmission electron micrograph for each group of AgNP solutions. The molar concentrations of the synthesized aqueous solution were calculated according to the size and absorbance of AgNPs, by means of the procedure suggested by Zhang *et al.* [27]. The results are summarized in table 1. As stated, the surface charges of the AgNPs were determined and are summarized in table 1. 

***Antibacterial activity***

**Figure 3:**
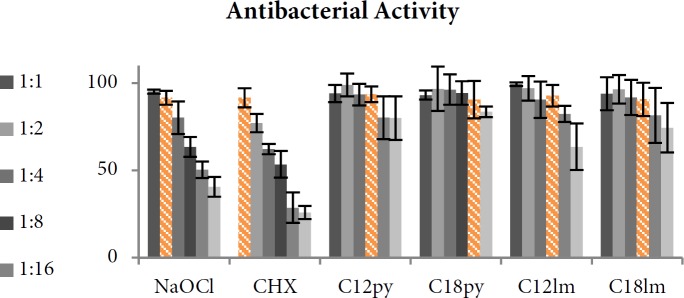
*Growth inhibition of E. faecalis as percentage of control group. The MIC*_90_ *(marked in orange color) is defined as the lowest concentration in which at least 90% of the bacterial growth was inhibited*

Im and Py resulted in 2.78% and 3.53% of bacterial growth inhibition. Figure 3 shows bacterial growth inhibition in exposure to different dilutions of experimental solutions. All tested AgNP solutions achieved MIC_90_ against *E. faecalis *in lower concentrations compared to CHX and NaOCl (Table 2). NaOCl had the highest MIC_90 _and C_12 _Py-AgNP solution had the lowest MIC value.

**Table 1.** The specifications of the synthesized AgNPs

**AgNPs Coating**	**λ**_max_ ** (nm)**	**A**_max_	**Average size (nm)**	**Zeta**	**Zeta/Radius**	**Concentration (M/L)**
**C**_12 _ **Py**	418	0.467	18.49 nm	+25.0	1.35	6.5×10^−^^9^
**C**_18 _ **Py**	406	1.075	6.71 nm	+57.6	8.58	1.37×10^−^^9^
**C**_12 _ **Im**	397	0.887	9.0 nm	+50.0	5.55	5.7×10^−^^8^
**C**_18 _ **Im**	394	1.21	8.6 nm	+58.2	6.76	6.5×10^−^^8^

**Table 2:** The MIC_90_ of the experimented solutions

**Solution**	**NaOCl**	**CHX**	**C**_12 _ **Py**	**C**_18 _ **Py**	**C**_12 _ **Im**	**C**_18 _ **Im**
**MIC**_90_ **(M/L)**	3.35×10^−1^	4×10^−3^	8.1x10^10^	8.5x10^-9^	7.1x10^-9^	8.1x10^-9^
**Cell viability (%)**	37.7849	3.26494	97.6678	99.1489	106.613	102.099

***Cytocompatibility assessment***

**Figure 4:**
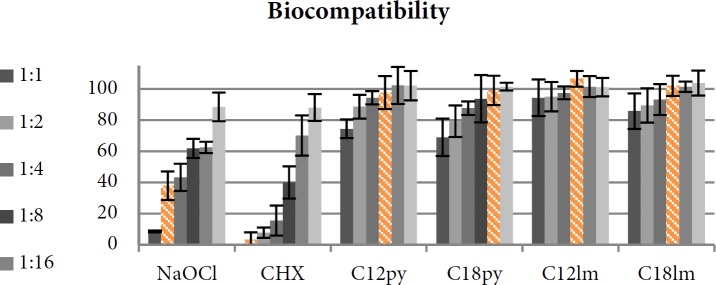
Viability of the fibroblast cells when exposed to different dilutions of each solution (The MIC is marked in orange color and is defined as the lowest concentration in which at least 90% of the bacterial growth was inhibited

The survival percentage of L929 fibroblasts when treated with different dilutions of the experimental solutions comes in figure 4. Im and Py resulted in 0.18% and 3.37% of toxicity to fibroblasts. CHX and NaOCl were more toxic to fibroblasts than all AgNP solutions.

One-Way ANOVA compared the viability of fibroblasts exposed to MICs of experimental solutions (Table 2) and reported a significant difference between AgNPs compared to NaOCl and CHX (*P*=0.00). The biocompatibility of NaOCl and CHX was significantly lower than all other tested solutions. There was no significant difference between any two AgNP solutions (*P*>0.05).

C_12 _Im AgNP had the highest biocompatibility regarding that 94.46% of fibroblasts survived while exposed to the highest experimented concentration (5.7×10^-8^M/L).

Two-way ANOVA was performed to evaluate the effects of the length of the alkyl chain and the type of ionic liquid separately on biocompatibility of AgNPs in different concentrations. There was a significant difference between AgNPs with Im coating compared to those with Py (*P*=0.024) but there was no significant difference between AgNPs with different alkyl chain lengths (*P*=0.167).

## Discussion

Regarding the favourable promises of AgNPs, there is an increasing tendency to use them as disinfectants in endodontics. Besides, finding an approach to enhance the properties of these particles to make them suitable for endodontic applications is still under investigation. Previously we exhibited that the surface charge of AgNPs was a significant factor in their bactericidal activity [24]. Further, we reported that the positively charged imidazolium-coated AgNPs have a high level of antimicrobial activity against a panel of microorganisms including *E. faecalis *along with cytocompatibility with fibroblast cells [14, 24]. In this research, the study was designed to evaluate the effects of variations in molecular structure including capping agents and the alkyl chains on the characteristics of AgNPs. Therefore, positively charged AgNPs capped by two different ionic liquids (Im and Py) with two different alkyl chain lengths (C_12_ and C_18_) were synthesized and compared with NaOCl and CHX as two of the most widely used irrigants in endodontics.

To standardize the comparisons, the surface charge to radius ratio of each nanoparticle was calculated. The evaluation of antibacterial activity revealed that all tested AgNPs had MICs remarkably lower than NaOCl and CHX. All AgNPs kept antibacterial potency higher than 90% killing in concentrations around 10^-9 ^M/L while this level of activity was achieved for CHX and NaOCl at concentrations of 10^-1^ and 10^-3^M/L respectively. Furthermore, the 1:1 concentration of C_12 _Im particles had a stronger antibacterial effect against *E. faecalis *than NaOCl as the most commonly used endodontic disinfectant which shows that this solution is capable to be used in clinical practice.

The excellent antimicrobial activity of AgNP solutions can be explained by the antibacterial properties of silver accompanied with presence of cationic molecules on the surface of NPs [28, 29]. The carboxyl, phosphate and amino groups present on the cellular membrane of gram-positive bacteria dictate negative charge of the microorganisms. Thus, the positive charge of ionic liquid-protected AgNPs could interact with the negatively charged microbial cell walls leading to alterations such as change in cell wall permeability, formation of pores and leakage of intracellular components, which can result in the extermination of the microorganism. This study further confirms previous findings demonstrating excellent potency of Im based AgNPs against a panel of microorganisms [24]. In addition, Patil *et al.* [30] demonstrated that AgNPs coated with 1-(dodecyl) 2 amino-pyridinium bromide had a high level of antibacterial activity.

**Figure 5:**
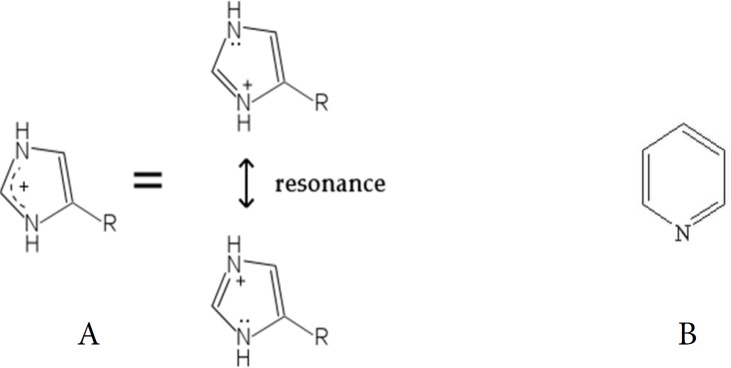
. A) Pyridinium (Py); B) Imidazolium (Im); Resonance in Py molecule contributes to an increase in the charge density while no resonance can be detected on Im molecule

The lowest and the highest MICs against *E. faecalis *belonged to C_12 _Py-coated AgNPs and NaOCl respectively. C_12 _Py-coated AgNPs had the lowest MIC value amongst all examined solutions. The ionic liquids were used as stabilizers as well as capping agents for AgNPs. They indicated the chemical functionality of AgNPs. The higher antibacterial activity of Py- coated AgNPs than Im-coated ones can be interpreted by the dissimilarity in charge distribution on the cationic part of the molecules. Therefore, the presence of charge resonance in Py’s molecular structure which causes higher charge density (Figure 5) may lead to higher antibacterial activity in Py-coated AgNPs. According to this finding, the type of ionic liquids was a more important factor in antibacterial activity than the alkyl chain length.

Regarding the clinical application of NPs, nanotoxicity is also an important issue remains to be considered. Therefore, MTT assay was employed to compare the cytotoxicity of the experimental solutions against L929 fibroblasts. This method is basically a cell culture technique which is quick, reliable, inexpensive and useful for the evaluation of cytocompatibility of the new medical materials. An L929 cell line was selected, given it is a well-characterized cell model and has been previously used to assess the cytotoxic effects of dental materials [31-35].

In the experimental conditions of the current study, NaOCl and CHX were significantly more cytotoxic than all tested AgNPs, both in their conventional concentrations and in their MICs. The highest cytotoxicity was reported for CHX at its MIC and only 3% of the cells survived when exposed to this solution.

Consistent with previous studies, our findings demonstrated that the toxic effect of AgNPs on L929 mouse fibroblasts is concentration dependent [36-38].

C_12 _Im coating of nanosilver showed the lowest toxicity to mouse fibroblasts, at its full concentration as well as its MIC. Irrespective to the alkyl chain length, Py-coated AgNPs had more cytotoxicity than Im-coated ones. Py-type compounds have previously been reported to be more cytotoxic than Im polymers [39, 40]. Likewise, Zhang *et al.* [41] reported that Im polymeric NPs did not show cytotoxic effects on eukaryotic cells.

The differences in charge distribution and the presence charge resonance can play the same role in nontoxicity as they did in antibacterial activity. However, there are different mechanisms effective in toxicity against bacteria and human cells. Although AgNPs can enter both prokaryotic and eukaryotic cells, it seems that cellular antioxidant mechanisms in eukaryotic cells protect them from possible oxidative damages [42]. Also there are differences in the amount of particle uptake between different cell types. This is necessary to make a balance between the desirable and undesirable effects of the presence of Py in AgNP structure. Considering that, both Im and Py coated AgNPs show antibacterial effects significantly higher than conventional root canal irrigants used in endodontics. The higher biocompatibility of Im-based solutions may bring them to higher priority for *in vivo* application. However, the 10 fold lower concentration of C_12 _Py-AgNP at its MIC that leads to lower amount of silver substance consumed in the process of synthesis makes it more cost-effective for clinical application.

Variations in the alkyl chain length from 12 carbon atoms to 18 had no significant impact on the bioactivity of AgNPs as well as their antibacterial effects. However, regarding to the presence of evidence on the effect of alkyl chain structure on the properties of the ionic liquids, further investigation is suggested to test higher ranges of variety in the number of carbon atoms and the molecular structure of this chain to brighten the role of this factor in bioactivity of ionic polymer-based agents as well as AgNPs. 

## Conclusion

The type of ionic liquid coatings and the length of alkyl chain can impact the bioactivity of AgNPs. Positively charged ionic liquid-protected AgNP solutions had excellent antibacterial activity against *E. faecalis* in very lower concentrations compared to NaOCl and CHX and exhibited higher cytocompatibility.
